# A guide to selecting high-performing antibodies for Syntenin-1 (O00560) for use in western blot, immunoprecipitation, and immunofluorescence

**DOI:** 10.12688/f1000research.183125.1

**Published:** 2026-06-26

**Authors:** Vera Ruíz Moleón, Charles Alende, Maryam Fothouhi, Sara González Bolívar, Riham Ayoubi, Vincent Francis, Peter S McPherson, Carl Laflamme

**Affiliations:** 1 Department of Neurology and Neurosurgery, Structural Genomics Consortium, The Montreal Neurological Institute, McGill University, Montreal, Canada

**Keywords:** UniProt ID O00560, SDCBP, Syntenin-1, Syndecan-binding protein 1, Melanoma differentiation-associated protein 9, MDA-9, antibody characterization, antibody validation, western blot, immunoprecipitation, immunofluorescence

## Abstract

Syntenin-1 is the Syndecan-binding protein 1 and a PDZ domain–containing adaptor protein that regulates diverse cellular processes through its interactions with transmembrane receptors, cytoskeletal components, and signaling molecules. Here we have characterized twelve Syntenin-1 commercial antibodies for western blot, immunoprecipitation, and immunofluorescence using a standardized experimental protocol based on comparing read-outs in knockout cell lines and isogenic parental controls. These studies are part of a larger, collaborative initiative seeking to address antibody reproducibility issues by characterizing commercially available antibodies for human proteins and publishing the results openly as a resource for the scientific community. While the use of antibodies and protocols vary between laboratories, we encourage readers to use this report as a guide to select the most appropriate antibodies for their specific needs.

## Introduction

Syntenin-1, encoded by *SDCBP* the gene, is a multifunctional PDZ-domain adaptor protein that integrates membrane dynamics with intracellular signaling. It organizes signalling complexes by binding to Syndecan and other partners, influencing cell membrane structure and trafficking. ^1^ Dysregulation of *SDCBP* expression has been increasingly associated with pathological phenotypes, most notably in cancer, where it promotes tumor cell survival, invasion, metastasis, and therapeutic resistance by modulating pathways such as Src, FAK, and Wnt/β-catenin. ^2^ RNA-seq data also reveals an increase of *SDCBP* levels in the brains of Alzheimer’s Disease patients when compared to controls. ^3^

This research is part of a broader collaborative initiative in which academics, funders and commercial antibody manufacturers are working together to address antibody reproducibility issues by characterizing commercial antibodies for human proteins using standardized protocols, and openly sharing the data. ^4^ It consists of identifying human cell lines with adequate target protein expression and the development/contribution of equivalent knockout (KO) cell lines, followed by antibody characterization procedures using most commercially available renewable antibodies against the corresponding protein. ^4^ Here we characterized twelve commercial Syntenin-1 antibodies, selected and donated by participant antibody manufacturers, for use in western blot, immunoprecipitation, and immunofluorescence (also referred to as immunocytochemistry), enabling biochemical and cellular assessment of Syntenin-1 properties and function.

The authors do not engage in result analysis or offer explicit antibody recommendations. Our primary aim is to deliver top-tier data to the scientific community, grounded in Open Science principles. This empowers experts to interpret the characterization data independently, enabling them to make informed choices regarding the most suitable antibodies for their specific experimental needs. Guidelines on how to interpret antibody characterization data found in this study are featured on the YCharOS gateway ^5^ and in Table 4 of this data note. ^4^

**Table 1.** Summary of the cell lines used.

Institution	Catalog number	RRID (Cellosaurus)	Cell line	Genotype
Horizon Discovery	C631	CVCL_Y019	HAP1	WT

**Table 2.** Summary of the Syntenin-1 antibodies tested.

Company	Catalog number	Lot number	RRID (Antibody Registry)	Clonality	Clone ID	Host	Concentration (μg/μL)	Vendors recommended applications
Abcam	ab133267 **	1027828–20	AB_11160262	recombinant mono	EPR8102	rabbit	0.59	Wb, IP, IF
ABclonal	A5497 **	4000001410	AB_2863506	recombinant mono	ARC1410	rabbit	0.80	Wb, IF
Aviva Systems Biology	ARP44535_T100	QC14970–20071116	AB_938445	polyclonal	-	rabbit	1.00	Wb
Aviva Systems Biology	ARP44537_P050	QC14971–100705	AB_10644746	polyclonal	-	rabbit	0.50	Wb
Bio-Techne (Novus Biologicals)	NBP3–26333 **	N0802W	AB_3638522	recombinant mono	19C1	rabbit	0.10	Wb, IF
Cell Signaling Technology	27964 **	1	AB_3698322	recombinant mono	E2I9L	rabbit	0.03	Wb, IP, IF
GeneTex	GTX108391	39799	AB_1951833	polyclonal	-	rabbit	1.00	Wb, IP, IF
GeneTex	GTX108470	39806	AB_2037949	polyclonal	-	rabbit	0.78	Wb, IF
GeneTex	GTX634154 *	42779	AB_2888404	monoclonal	GT1523	mouse	1.00	Wb, IF
Thermo Fisher Scientific	MA5–34819 **	AC4649684B	AB_2848727	recombinant mono	JE40–72	rabbit	1.00	Wb, IP, IF
Thermo Fisher Scientific	MA5–35685 **	AC4650224A	AB_2849585	recombinant mono	3J5O6	rabbit	0.80	Wb, IF
Thermo Fisher Scientific	MA5–53244 **	AC4649692	AB_3247717	recombinant mono	23GB2185	rabbit	0.80	Wb, IF

Wb = western blot; IP = immunoprecipitation; IF = immunofluorescence,

** = recombinant antibody,

* = monoclonal antibody

**Table 3.** Table of secondary antibodies used.

Company	Secondary antibody	Catalog number	RRID (Antibody Registry)	Clonality	Concentration (μg/μL)	Working concentration (μg/mL)
Proteintech	HRP-Goat Anti-Rabbit Antibody (H + L)	RGAR001	AB_3073505	recombinant polyclonal	1.0	0.05
Proteintech	HRP-Goat Anti-Mouse Antibody (H + L)	RGAM001	AB_3068333	recombinant polyclonal	1.0	0.5
Cell Signaling Technology	Protein A, HRP conjugate	12291	NA	polyclonal	0.125	0.5
MilliporeSigma	Protein A, HRP conjugate	18–160	NA	polyclonal	1.0	2.0
Proteintech	CoraLite Plus 555-Goat Anti-Rabbit Antibody (H + L)	RGAR003	AB_3073507	recombinant polyclonal	0.5	0.5
Proteintech	CoraLite Plus 555-Goat Anti-Mouse Antibody (H + L)	RGAM003	AB_3068539	recombinant polyclonal	0.5	0.5

**Table 4.** Illustrations to assess antibody performance in all western blot, immunoprecipitation and immunofluorescence.

Western blot	Immunoprecipitation	Immunofluorescence
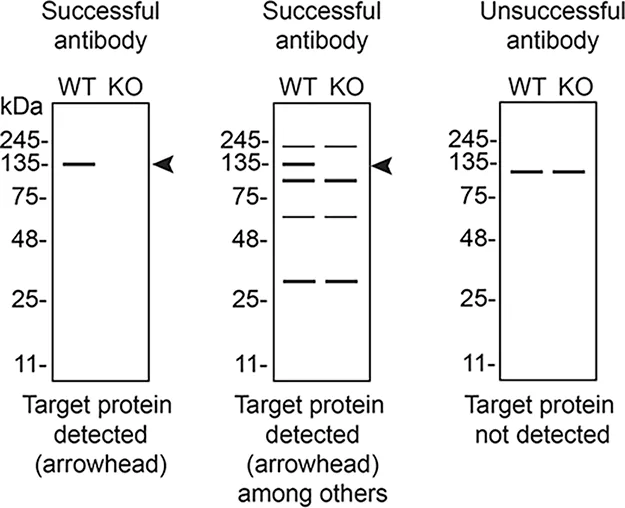	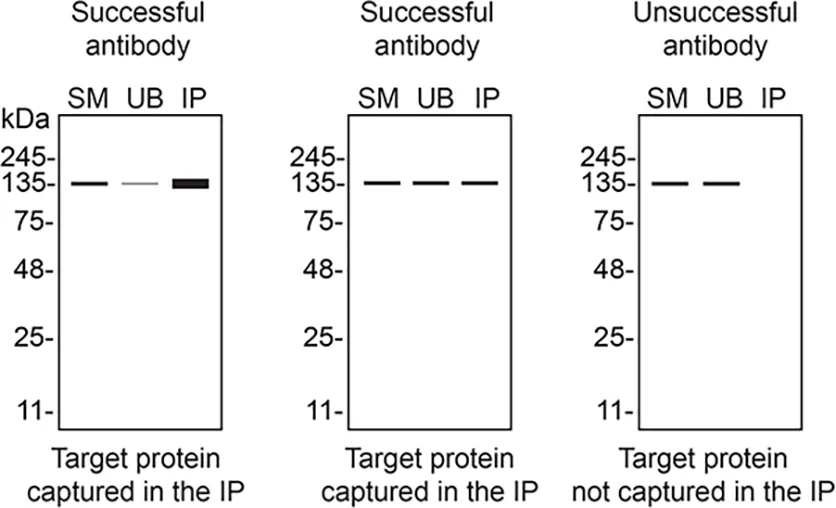	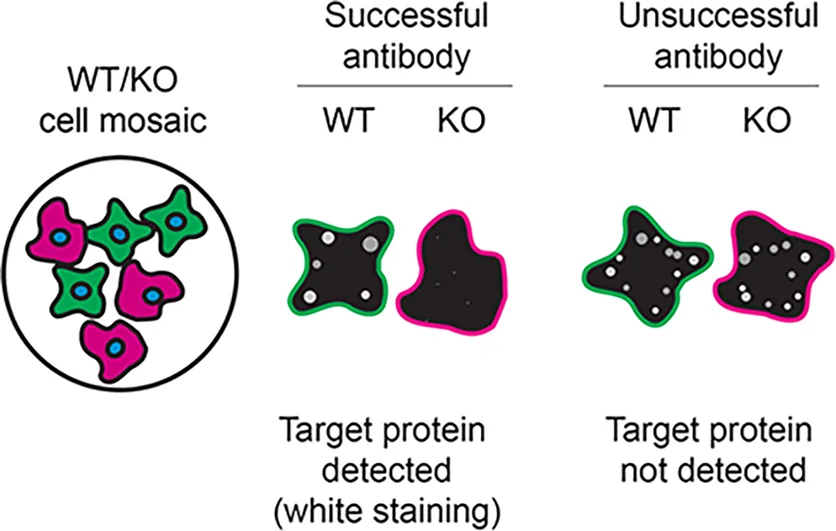

This table has been reproduced with permission from Ayoubi et al., Elife, 2023 ^9^

## Results and discussion

Our standard protocol involves comparing readouts from wild type (WT) and KO cell lines. ^6^ In the absence of commercially available KO cell lines, siRNA technology can be employed to knockdown (KD) the target gene. ^7, 8^ To determine which cell line demonstrates high expression of *SDCBP* and thus be appropriate for KD, we examined the DepMap (Cancer Dependency Map Portal, RRID: SCR_017655) transcriptomics database to identify cell lines that express the target at levels greater than 2.5 log _2_ (transcripts per million “TPM” + 1), which we have found to be a suitable cut-off. ^9^ As a result, HAP1 was identified, and a non-targeting control siRNA pool was used to treat HAP1 control (ctrl) cells, while *SDCBP* was KD using a pool of siRNA targeting this gene.

**Figure 1.**
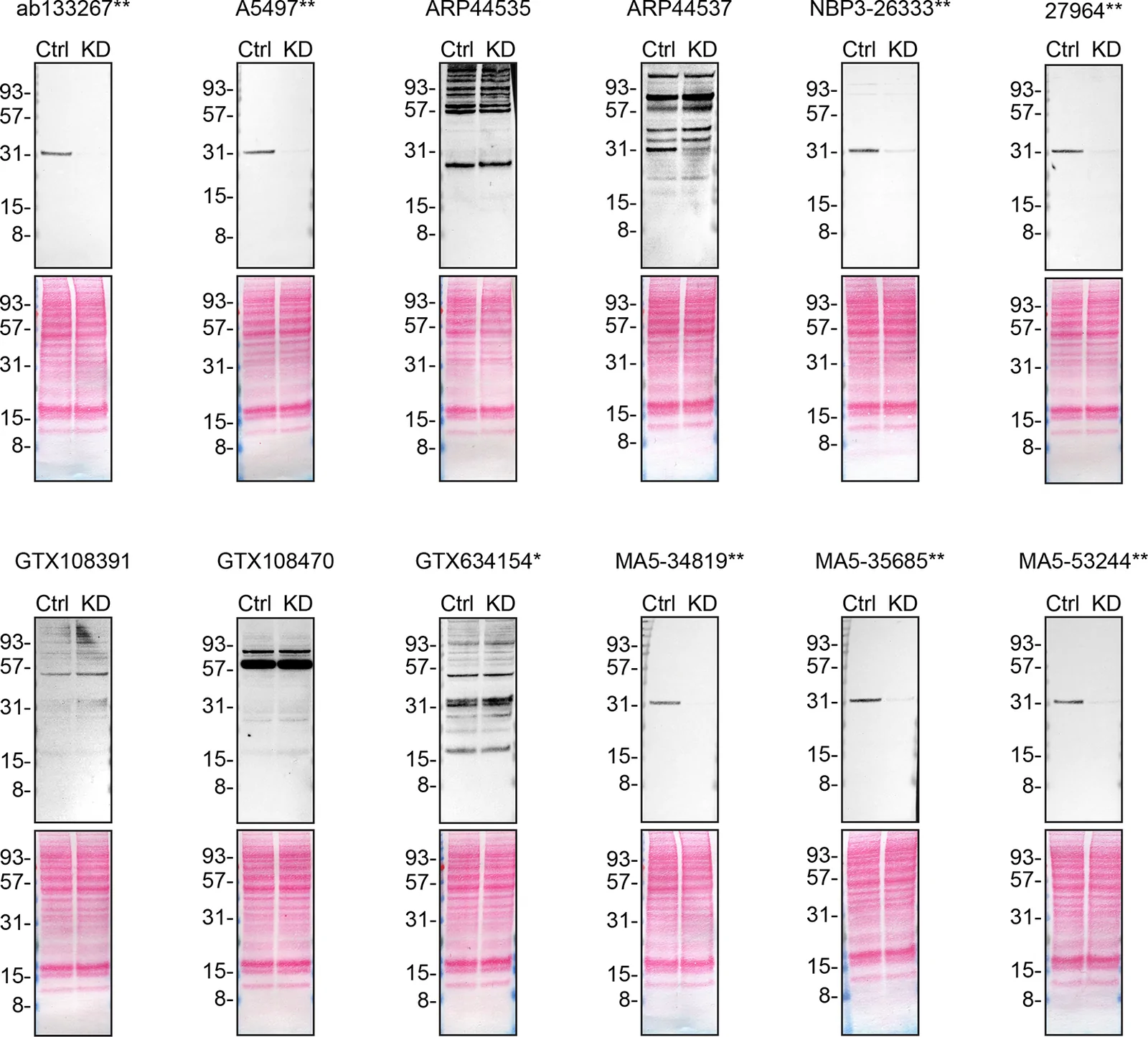
Syntenin-1 antibody screening by western blot on lysate. Lysates of HAP1 ctrl and *SDCBP* KD were prepared, and 30 μg of protein were processed for western blot with the indicated Syntenin-1 antibodies. The Ponceau stained transfers of each blot are presented to show equal loading of ctrl and KD lysates and protein transfer efficiency from the acrylamide gels to the nitrocellulose membrane. Antibody dilutions were chosen according to the recommendations of the antibody supplier. Antibody dilutions used: ab133267** at 1/1000; A5497** at 1/500; ARP44535_T100 at 1/1000; ARP44537_P050 at 1/500; NBP3–26333** at 1/500; 27964** at 1/1000; GTX108391 at 1/500; GTX108470 at 1/500; GTX634154* at 1/500; MA5–34819** at 1/2000; MA5–35685** at 1/500; MA5–53244** at 1/1000. Predicted band size: 32.5 kDa. ** = recombinant antibody, * = monoclonal antibody.

**Figure 2.**
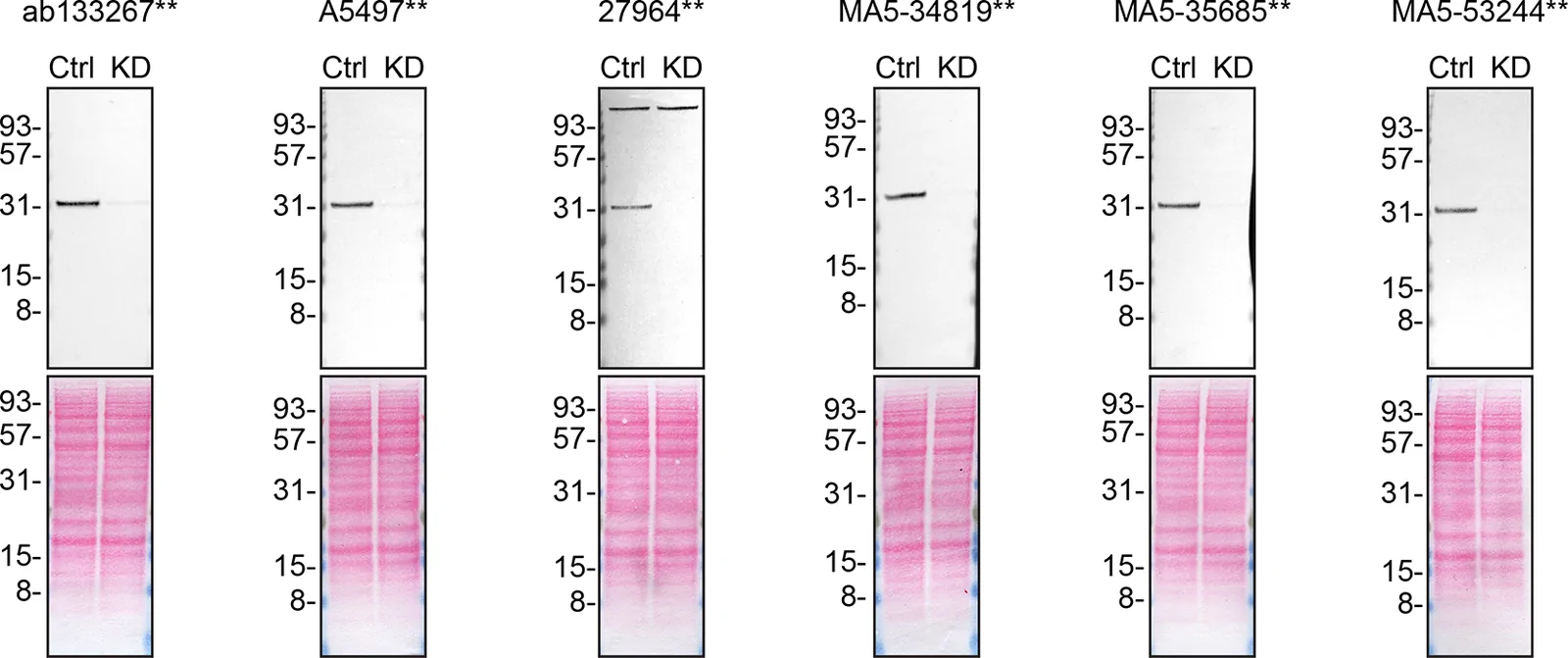
Syntenin-1 antibody screening by western blot on culture medium. Culture media from HAP1 ctrl and *SDCBP* KD were collected, and 30 μg of protein were processed for western blot with the indicated Syntenin-1 antibodies. The Ponceau stained transfers of each blot are presented to show equal loading of ctrl and KD samples and protein transfer efficiency from the acrylamide gels to the nitrocellulose membrane. Antibody dilutions used: ab133267** at 1/1000; A5497** at 1/500; 27964** at 1/1000; MA5–34819** at 1/2000; MA5–35685** at 1/500; MA5–53244** at 1/1000. Predicted band size: 32.5 kDa. ** = recombinant antibody, * = monoclonal antibody.

To screen the twelve antibodies by western blot, ctrl and *SDCBP* KD protein lysates were ran on SDS-PAGE, transferred onto nitrocellulose membranes, and then probed with all twelve Syntenin-1 antibodies in parallel ( Figure 1). To detect secreted Syntenin-1, six renewable recombinant antibodies were screened by western blot with HAP1 ctrl and *SDCBP* KD proteins from culture medium ran on SDS-PAGE, transferred onto nitrocellulose membranes, and then probed with the six Syntenin-1 antibodies in parallel ( Figure 2).

**Figure 3.**
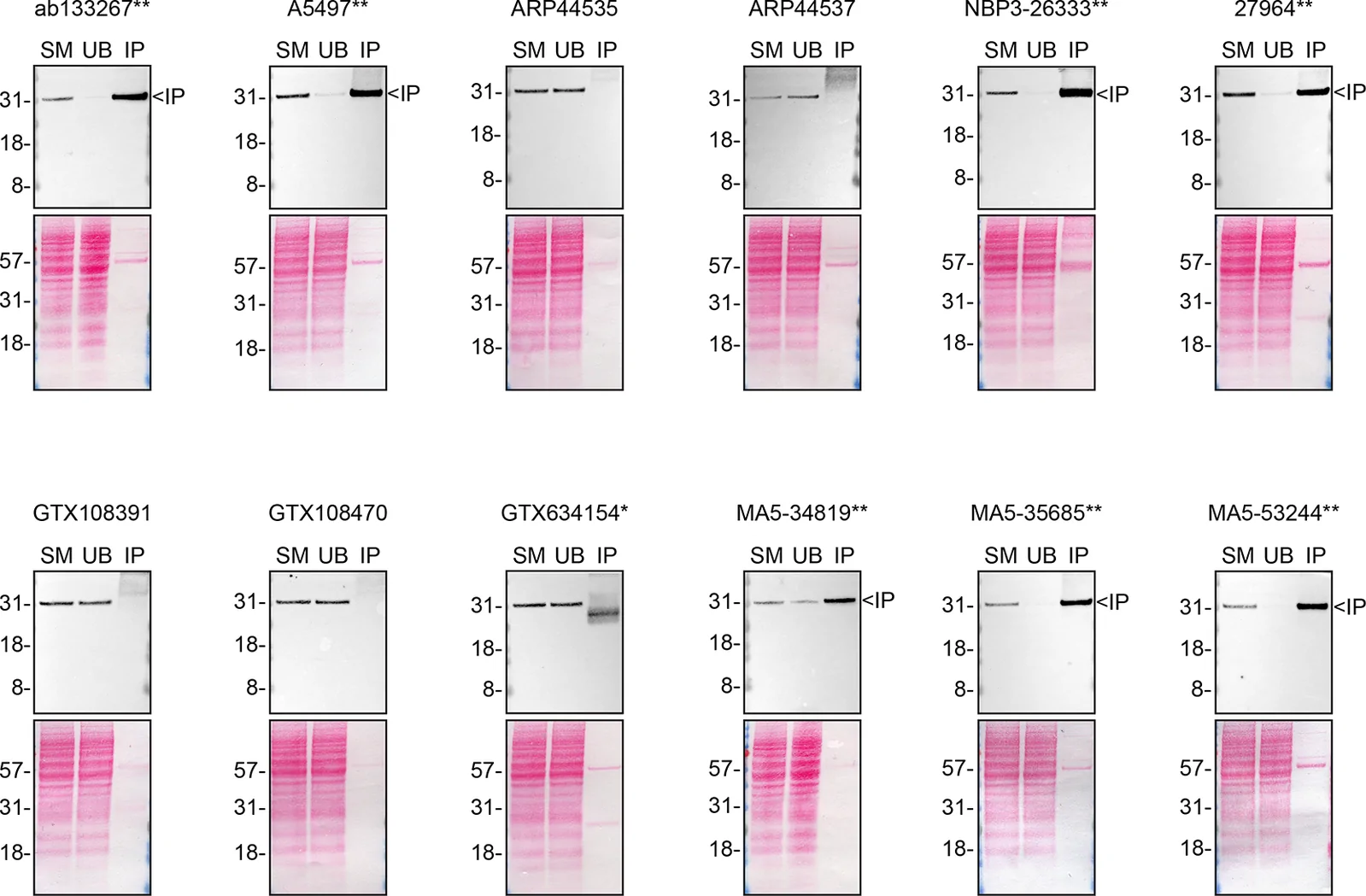
Syntenin-1 antibody screening by immunoprecipitation on lysate. HAP1 WT lysates were prepared, and immunoprecipitation was performed for 1 h using 0.5 mg of lysate and 2.0 μg of the indicated Syntenin-1 antibodies pre-coupled to Dynabeads protein A or protein G. Samples were washed and processed for western blot with the Syntenin-1 antibody MA5–35685** used at 1/500. The Ponceau stained transfers of each blot are shown. SM = 6% starting material; UB = 6% unbound fraction; IP = immunoprecipitate, ** = recombinant antibody, * = monoclonal antibody.

**Figure 4.**
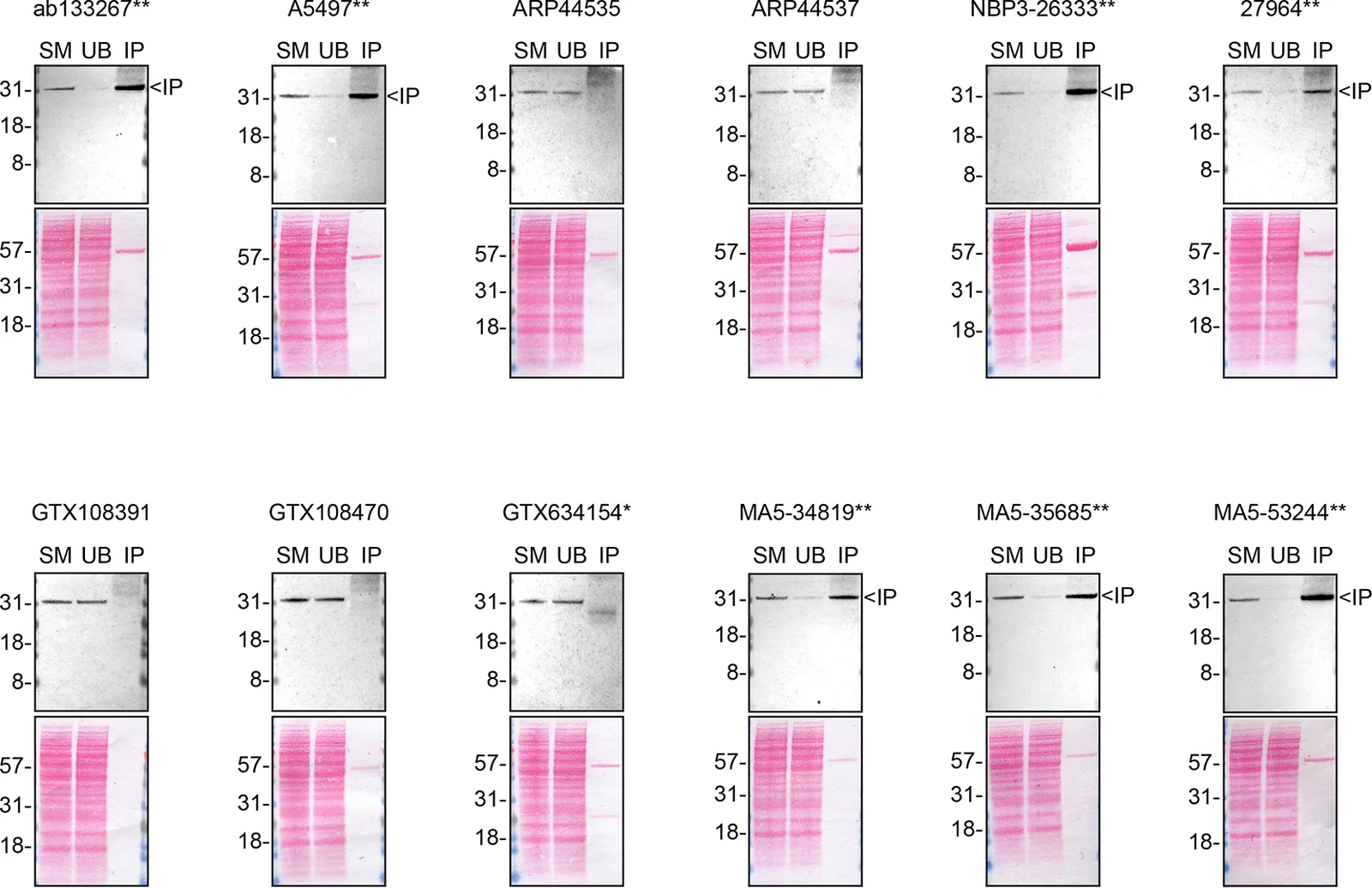
Syntenin-1 antibody screening by immunoprecipitation on culture medium. Culture medium was collected from HAP1 WT, and immunoprecipitation was performed for 1 h using 0.35 mg of protein and 2.0 μg of the indicated Syntenin-1 antibodies pre-coupled to Dynabeads protein A or protein G. Samples were washed and processed for western blot with the anti-Syntenin-1 antibody MA5–34819** diluted at 1/1000. The Ponceau stained transfers of each blot are shown. SM = 6% starting material; UB = 6% unbound fraction; IP = immunoprecipitate** = recombinant antibody, * = monoclonal antibody.

We then assessed the capability of all twelve antibodies to capture Syntenin-1 from HAP1 protein extracts using immunoprecipitation techniques, followed by western blot analysis. For the immunoblot step, a specific Syntenin-1 antibody identified previously (refer to Figure 1) was selected. Equal amounts of the starting material (SM) and the unbound fractions (UB), as well as the whole immunoprecipitate (IP) eluates were separated by SDS-PAGE ( Figure 3). The twelve antibodies were also tested for their ability to capture Syntenin-1 from HAP1 culture medium using the same immunoprecipitation technique ( Figure 4).

**Figure 5.**
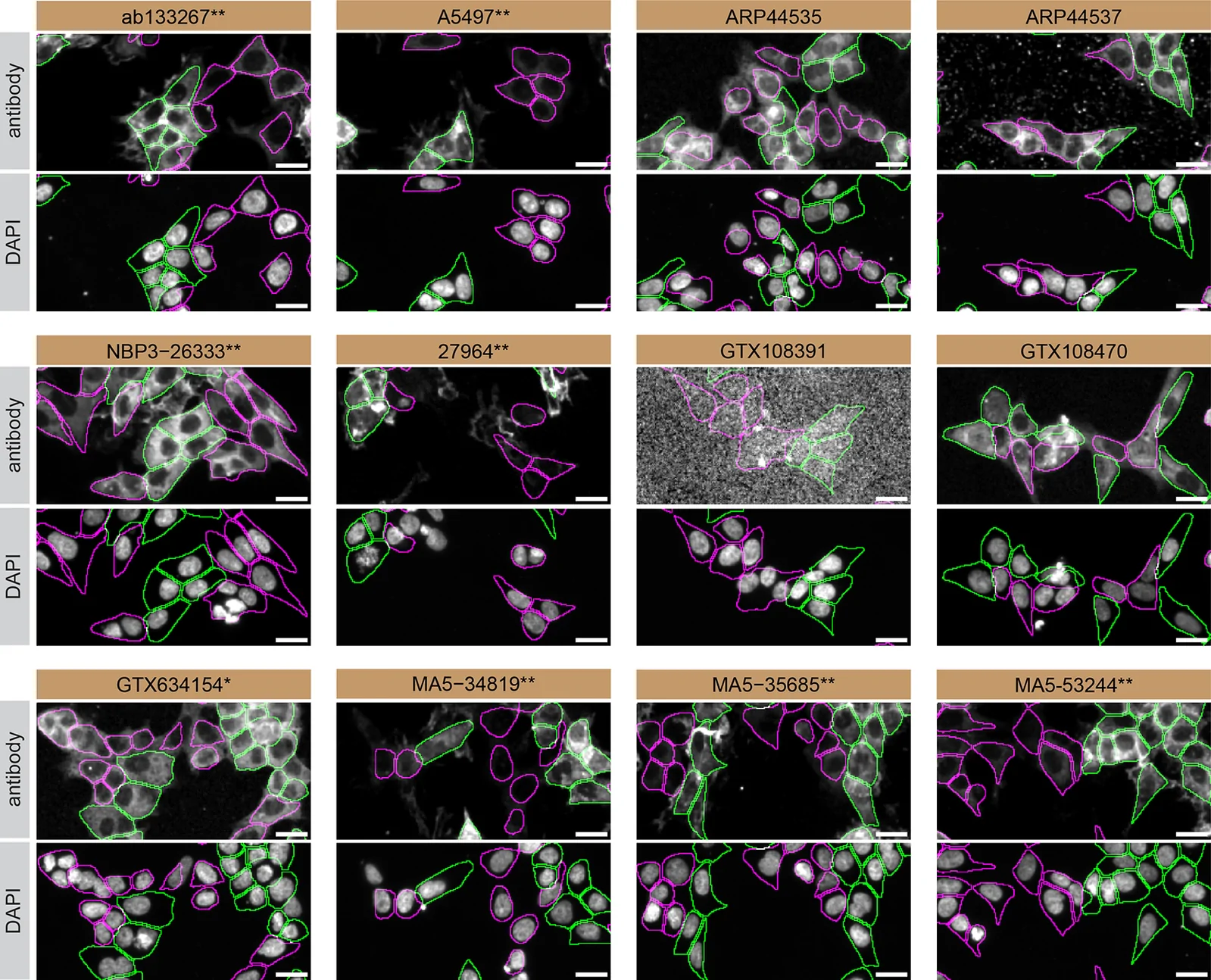
Syntenin-1 antibody screening by immuofluorescence. HAP1 ctrl and *SDCBP* KD cells were labelled with a green or a far-red fluorescent dye, respectively. Ctrl and KD cells were mixed and plated to a 1:1 ratio in a 96-well plate with optically clear flat-bottom. Cells were stained with the indicated Syntenin-1 antibodies and with the corresponding fluor 555 coupled secondary antibody including DAPI. Acquisition of the blue (nucleus-DAPI), green (ctrl), red (antibody staining) and far-red (KD) channels was performed. Representative images of the blue and red (grayscale) channels are shown. Ctrl and KD cells are outlined with green and magenta dashed line, respectively. When an antibody was recommended for immunofluorescence by the supplier, we tested it at the recommended dilution. The rest of the antibodies were tested at 1 and 2 μg/ml, and the final concentration was selected based on the detection range of the microscope used and a quantitative analysis not shown here. Antibody dilutions used: ab133267** at 1/500; A5497** at 1/800; ARP44535_T100 at 1/500; ARP44537_P050 at 1/500; NBP3–26333** at 1/100; 27964** at 1/100; GTX108391 at 1/500; GTX108470 at 1/500; GTX634154* at 1/500; MA5–34819** at 1000/; MA5–35685** at 1/800; MA5–53244** at 1/1000. Bars = 10 μm. ** = recombinant antibody, * = monoclonal antibody.

For immunofluorescence, the twelve antibodies were screened using a mosaic strategy. First, HAP1 ctrl and *SDCBP* KD cells were labelled with different fluorescent dyes in order to distinguish the two cell lines, and the Syntenin-1 antibodies were evaluated. Both ctrl and KD lines imaged in the same field of view to reduce staining, imaging and image analysis bias ( Figure 5). Quantification of immunofluorescence intensity in hundreds of ctrl and KD cells was performed for each antibody tested, and the images presented in Figure 5 are representative of this analysis. ^4^

In conclusion, we have screened twelve Syntenin-1 commercial antibodies by western blot, immunoprecipitation, and immunofluorescence by comparing the signal produced by the antibodies in human HAP1 ctrl and *SDCBP* KD cells. Western blot and immunoprecipitation were performed in both lysate and conditioned medium. To assist users in interpreting antibody performance, Table 4 outlines various scenarios in which antibodies may perform in all three applications. ^9^ High-quality and renewable antibodies that successfully detect Syntenin-1 were identified in all applications. Researchers who wish to study Syntenin-1 in a different species are encouraged to select high-quality antibodies, based on the results of this study, and investigate the predicted species reactivity of the manufacturer before extending their research.

## Limitations

Inherent limitations are associated with the antibody characterization platform used in this study. Firstly, the YCharOS project focuses on renewable (recombinant and monoclonal) antibodies and does not test all commercially available Syntenin-1 antibodies. YCharOS partners provide approximately 80% of all renewable antibodies, but some top-cited polyclonal antibodies may not be available through these partners. We encourage readers to consult vendor documentation to identify the specific antigen each antibody is raised against, where such information is available.

Secondly, the YCharOS effort employs a non-biased approach that is agnostic to the protein for which antibodies have been characterized. The aim is to provide objective data on antibody performance without preconceived notions about how antibodies should perform or the molecular weight that should be observed in western blot. As the authors are not experts in Syntenin-1, only a brief overview of the protein’s function and its relevance in disease is provided. Syntenin-1 experts are invited to analyze and interpret observed banding patterns in western blots and subcellular localization in immunofluorescence.

Thirdly, YCharOS experiments are not performed in replicates primarily due to the use of multiple antibodies targeting various epitopes. Once a specific antibody is identified, it validates the protein expression of the intended target in the selected cell line, confirms the lack of protein expression in the KO cell line and supports conclusions regarding the specificity of the other antibodies. All experiments are performed using master mixes, and meticulous attention is paid to sample preparation and experimental execution. In IF, the use of two different concentrations serves to evaluate antibody specificity and can aid in assessing assay reliability. In instances where antibodies yield no signal, a repeat experiment is conducted following titration. Additionally, our independent data is performed subsequently to the antibody manufacturers internal validation process, therefore making our characterization process a repeat.

Lastly, as comprehensive and standardized procedures are respected, any conclusions remain confined to the experimental conditions and cell line used for this study. The use of a single cell type for evaluating antibody performance poses as a limitation, as factors such as target protein abundance significantly impact results. Additionally, the use of cancer cell lines containing gene mutations poses a potential challenge, as these mutations may be within the epitope coding sequence or other regions of the gene responsible for the intended target. Such alterations can impact the binding affinity of antibodies. This represents an inherent limitation of any approach that employs cancer cell lines.

## Method

The standardized protocols used to carry out this KO cell line-based antibody characterization platform was established and approved by a collaborative group of academics, industry researchers and antibody manufacturers. The detailed materials and step-by-step protocols used to characterize antibodies in western blot, immunoprecipitation and immunofluorescence are openly available on Protocols.io ( protocols.io/view/a-consensus-platform-for-antibody-characterization ). ^4^ Brief descriptions of the experimental setup used to carry out this study can be found below.

### Cell lines and antibodies

The cell lines, primary and secondary antibodies used in this study are listed in Table 1, 2, and 3, respectively. To ensure consistency with manufacturer recommendations and account for proprietary formulations (where antibody concentrations are not disclosed), antibody usage is reported as dilution ratios rather than absolute concentrations. To facilitate proper citation and unambiguous identification, all cell lines and antibodies are referenced with their corresponding Research Resource Identifiers (RRIDs). ^10, 11^ All cell lines used in this study were regularly tested for mycoplasma contamination and were confirmed to be mycoplasma-free.

To knockdown *SDCBP*, HAP1 cells were treated with the *SDCBP* SMARTPool from Horizon Discovery (cat. Number L-008270-00) for five days. HAP1 control cells were treated with the ON-TARGETplus Non-targeting Control Pool, Horizon Discovery (cat. Number D-001810-10). Lipofectamine RNAiMAX (Thermo Fisher Scientific, cat. Number 13778030) was used to transfect the siRNA following the manufacturer’s protocol.

### Antibody screening by western blot

*Lysate preparation*

HAP1 ctrl and *SDCBP* KD cells were collected in RIPA buffer (25 mM Tris-HCl pH 7.6, 150 mM NaCl, 1% NP-40, 1% sodium deoxycholate, 0.1% SDS) (Thermo Fisher Scientific, cat. Number 89901) supplemented with 1x protease inhibitor cocktail mix (MilliporeSigma, cat. Number P8340). Lysates were sonicated briefly and incubated 30 min on ice. Lysates were spun at ~110,000 *x g* for 15 min at 4 °C and equal protein aliquots of the supernatants were analyzed by SDS-PAGE and western blot. BLUelf prestained protein ladder (GeneDireX, cat. Number PM008–0500) was used.

*Culture medium collection*

HAP1 ctrl and *SDCBP* KD cells were washed 3x with PBS 1x and starved for ~36 hrs (2 nights). Culture media were collected and centrifuged for 10 min at 500 x *g* to eliminate cells and larger contaminants, then for 10 min at 4500 x *g* to eliminate smaller contaminants. Culture media were then concentrated by centrifuging at 4000 x *g* for 30 min using the Amicon Ultra-15 Centrifugal Filter Units with a membrane NMWL of 10 kDa (MilliporeSigma cat. Number UFC901024). Culture media were finally supplemented with 1x protease inhibitor cocktail mix.

For both lysate and medium, western blots were performed with precast midi 10% Bis-Tris polyacrylamide gels (Thermo Fisher Scientific, cat. Number WG1201BOX) ran with MES SDS buffer (Thermo Fisher Scientific, cat. Number NP000202), loaded in LDS sample buffer (Thermo Fisher Scientific, cat. Number NP0008) with 1x sample reducing agent (Thermo Fisher Scientific, cat. Number NP0009) and transferred on nitrocellulose membranes. Proteins on the blots were visualized with Ponceau S staining (Thermo Fisher Scientific, cat. Number BP103–10) which is scanned to show together with individual western blot. Blots were blocked with 5% milk for 1 hr, and antibodies were incubated O/N at 4 °C with 5% milk in TBS with 0,1% Tween 20 (TBST) (Cell Signalling Technology, cat. Number 9997). Following three washes with TBST, membranes were incubated with the peroxidase conjugated secondary antibody diluted in TBST with 5% milk for 1 hr at room temperature followed by three washes with TBST. Membranes were incubated with Pierce ECL (Thermo Fisher Scientific, cat. Number 32106) or Clarity Western ECL Substrate (Bio-Rad, cat. Number 1705061) prior to detection with the iBright™ CL1500 Imaging System (Thermo Fisher Scientific, cat. Number A44240).

### Antibody screening by immunoprecipitation

*Immunoprecipitation with lysate*

Antibody-bead conjugates were prepared by adding 2 μg to 500 μl of Pierce IP Lysis Buffer from Thermo Fisher Scientific (cat. Number 87788) in a microcentrifuge tube, together with 30 μl of Dynabeads protein A- (for rabbit antibodies) or protein G- (for mouse antibodies) (Thermo Fisher Scientific, cat. Number 10002D and 10004D, respectively). All tubes were rocked for ~1 h at 4 °C followed by two washes to remove unbound antibodies.

HAP1 WT cells were collected in Pierce IP buffer (25 mM Tris-HCl pH 7.4, 150 mM NaCl, 1 mM EDTA, 1% NP-40 and 5% glycerol) supplemented with protease inhibitor. Lysates were rocked 30 min at 4 °C and spun at 110,000 *x g* for 15 min at 4 °C. 0.5 mL aliquots at 1.0 mg/mL of lysate were incubated with an antibody-bead conjugate for ~1 h at 4 °C. The unbound fractions were collected, and beads were subsequently washed three times with 1.0 ml of IP buffer and processed for SDS-PAGE and western blot on precast midi 10% Bis-Tris polyacrylamide gels.

*Immunoprecipitation with culture medium*

Culture media from HAP1 WT were collected as described in the western blotting section above. 0.35 mL aliquots at 1.0 mg/mL of culture medium were incubated with an antibody-bead conjugate for ~1 h at 4 °C. The unbound fractions were collected, and beads were subsequently washed three times with 1.0 mL of IP buffer and processed for SDS-PAGE and western blot on precast midi 10% Bis-Tris polyacrylamide gels.

### Antibody screening by immunofluorescence

HAP1 ctrl and *SDCBP* KD cells were labelled with a green and a far-red fluorescence dye, respectively (Thermo Fisher Scientific, cat. Number C2925 and C34565). The nuclei were labelled with DAPI (Thermo Fisher Scientific, cat. Number D3571) fluorescent stain. Ctrl and KD cells were plated on 96-well plate with optically clear flat-bottom (Perkin Elmer, cat. Number 6055300) as a mosaic and incubated for 24 hrs in a cell culture incubator at 37 °C, 5% CO _2_. Cells were fixed in 4% paraformaldehyde (PFA) (VWR, cat. Number 100503–917) in phosphate buffered saline (PBS) (Wisent, cat. Number 311–010-CL). Cells were permeabilized in PBS with 0,1% Triton X-100 (Thermo Fisher Scientific, cat. Number BP151–500) for 10 min at room temperature and blocked with PBS with 5% BSA, 5% goat serum (Gibco, cat. Number 16210–064) and 0.01% Triton X-100 for 30 min at room temperature. Cells were incubated with IF buffer (PBS, 5% BSA, 0,01% Triton X-100) containing the primary Syntenin-1 antibodies overnight at 4 °C. Cells were then washed 3 × 10 min with IF buffer and incubated with corresponding Alexa Fluor 555-conjugated secondary antibodies in IF buffer at a dilution of 1.0 μg/ml for 1 hr at room temperature with DAPI. Cells were washed 3 × 10 min with IF buffer and once with PBS.

Images were acquired on an ImageXpress micro confocal high-content microscopy system (Molecular Devices), using a 20x NA 0.94 air objective and scientific CMOS cameras, equipped with 395, 475, 555 and 635 nm solid state LED lights (lumencor Aura III light engine) and bandpass filters to excite DAPI, Cellmask Green, Alexa-555 and Cellmask Red, respectively. Images had pixel sizes of 0.68 x 0.68 microns, and a z-interval of 4 microns. For analysis and visualization, shading correction (shade only) was carried out for all images. Then, maximum intensity projections were generated using 3 z-slices. Segmentation was carried out separately on maximum intensity projections of Cellmask channels using CellPose 1.0, and masks were used to generate outlines and for intensity quantification. ^12^ Figures were assembled with Adobe Illustrator.

## Data Availability

Zenodo: Dataset for the Syntenin-1 antibody screening study < https://doi.org/10.5281/zenodo.20293661> Data are available under the terms of the Creative Commons Attribution 4.0 International license (CC-BY 4.0).
