# Supplementary material for: A guide to selecting high-performing antibodies for Syntenin-1 (O00560) for use in western blot, immunoprecipitation, and immunofluorescence
Source: F1000Res. 2026 Jun 26;15:1021. [Version 1] doi: 10.12688/f1000research.183125.1 (PMC13562940; doi:10.12688/f1000research.183125.1)
Supplement: Peer review history

# Reviewer report: Reviewer response for version 1

**DOI:** 10.5256/f1000research.202133.r508245

**Published:** 2026-09-04

**Authors:** Karen Bowman

**Affiliations:** 1 University of Leicester, Leicester, England, UK

The study involved characterisation of twelve commercial antibodies, from seven different companies, for the Syntenin-1 protein (Syndecan-binding protein 1), a PDZ-domain scaffold protein encoded by the *SDCBP* gene, for use in western blot, immunoprecipitation, and immunofluorescence. The study formed part of a wider YCharOS collaboration to objectively characterise commercial antibodies, which must be renewable (recombinant and monoclonal), for human proteins using standardised protocols with the intention of improving antibody reproducibility issues. As a Data Note, it allows the direct comparison of the performance of commercially available antibodies to the Syntenin-1 protein to aid scientists in choosing the most appropriate antibody for their studies. The Syntenin-1 protein is becoming of increasing interest as a biomarker and therapeutic target in oncology as well as in other areas such as inflammatory and immune-related disorders and this study would be pertinent to these fields.

 A committee of industry and academic representatives have endorsed the platform used, and the protocols were consistent with those in general use. The platform consisted of identification of a human cell line suitable for antibody characterisation studies i.e., with adequate levels of the Syntenin-1 protein to generate a measurable signal. Using the DepMap (Cancer Dependency Map Portal, RRID: SCR_017655) transcriptomics database, they found that the commercially available HAP1 cell line expresses *SDCBP* transcript at a level above the average range for cancer cells analysed, and therefore, was a logical choice for their study. Knockdown of the target gene using siRNA technology, with a pool of siRNAs, was used to provide appropriate negative control cells. This was used as no commercial KO in a cell line background that expresses adequate levels of Syntenin-1 was available. A non-targeting control siRNA pool was used to treat HAP1 control (ctrl) cells, which was the appropriate control for the assays presented.

 The final step was a series of antibody characterisation procedures, limited to the most common research uses of antibodies. The interpretation of the results is left to the reader, with the study providing unbiased guidance. However, it gave some indication of the optimum dilutions of commercial antibodies. Limitations of the study are clearly stated. Antibody detection of Syntenin-1 in both cell lysates and its excretion into cell culture medium was investigated in this study.

 A minor correction needed is that, in the introduction section, in the first sentence, “the” should go before *SDCBP.*

 One reservation I have is that in figure 5, the image of cells treated with GTX108391 has a very high background level of staining. This high background needs explanation in the text by the authors if the image is to be left in the article. The reader has no evidence from what is presented as to whether the high background is caused by the antibody, contamination, or an artifact, particularly as this is a one-off run. If the authors want to leave this image in the article, they should present what evidence they have that this background is due to the antibody or repeat the immunofluorescence assay for that antibody.

Are sufficient details of methods and materials provided to allow replication by others?

Yes

Is the rationale for creating the dataset(s) clearly described?

Yes

Are the datasets clearly presented in a useable and accessible format?

Yes

Are the protocols appropriate and is the work technically sound?

Yes

Reviewer Expertise:

Cell biology, DNA damage and repair, anti-cancer drug discovery and development, diagnostics

I confirm that I have read this submission and believe that I have an appropriate level of expertise to confirm that it is of an acceptable scientific standard.

# Reviewer report: Reviewer response for version 1

**DOI:** 10.5256/f1000research.202133.r498830

**Published:** 2026-07-22

**Authors:** Jieya Shao

**Affiliations:** 1 Washington University in St Louis, St. Louis, USA

In this article the authors systematically compared the performance and specificity of twelve commercially available anti-Syntenin-1 antibodies using three standard experimental approaches (western blot, immunoprecipitation, and immunofluorescence staining). This study is part of a large collaborative initiative to characterize commercial antibodies for different human proteins and offer unbiased guidance to researchers who can select the best antibodies for their specific experimental needs. As a Data Note, this paper successfully achieved its goal with carefully collected high-quality data, and the results are useful for the research community.

 Minor comments:

 1) On page 3, in the last paragraph, the sentence “Both ctrl and KD lines imaged in the same field of view…” seems to lack a verb. The authors may want to correct this by adding “were” (i.e., “Both ctrl and KD lines were imaged in the same field of view…”).

 2) It may provide more clarity to certain viewers by using full words instead of abbreviations such as ctrl and KD.

Are sufficient details of methods and materials provided to allow replication by others?

Yes

Is the rationale for creating the dataset(s) clearly described?

Yes

Are the datasets clearly presented in a useable and accessible format?

Yes

Are the protocols appropriate and is the work technically sound?

Yes

Reviewer Expertise:

cancer biology, DNA replication, chemotherapy resistance, DNA damage response

I confirm that I have read this submission and believe that I have an appropriate level of expertise to confirm that it is of an acceptable scientific standard.
